# Supplementary material for: Prevalence of bacteriologically confirmed pulmonary tuberculosis and associated risk factors: A community survey in Thirvallur District, south India
Source: PLoS One. 2021 Oct 5;16(10):e0247245. doi: 10.1371/journal.pone.0247245 (PMC8491886; doi:10.1371/journal.pone.0247245)
Supplement: Attachment — Submitted filename: Reply to reviewers comments 2.docx [file pone.0247245.s001.docx]

**Reply to Reviewers comments**

**Comments to the Author**

| **S. No** | **Comments** | **Reply** |
| --- | --- | --- |
|  | Reply to Reviewer #2 |  |
|  | In this manuscript, Dolla et al. summarized an epidemiology study on active pulmonary tuberculosis (PTB) at Tiruvallure district via cluster sampling method. The authors defined PTB according to positive smear test or bacterial culture test. Out of 62494 screened population, the authors determined a PTB prevalence rate as 307 per million population. The authors further reported positive correlation between PTB with decreased BMI, smoking, diabetes and elder population. The study overall is convincing, however the authors need to address the following issues | **-** |
| **1** | The BCG vaccination does not have protection judged by a p value of 0.792. Can the authors comment on the possible reasons? | Thank you for this valid question. We would like to clarify that it was documented that the protective efficacy of BCG was effective for children protecting from the severe forms of TB.  Reference: Subramani R, Datta M, Swaminathan S. Does effect of BCG vaccine decrease with time since vaccination and increase tuberculin skin test reaction? Indian J Tuberc 2015; 62(4): 226-9. |
| **2** | The authors applied p value test to justify the statistical significance between 2 groups. However, the authors need to specify the mathematical method and type-II error for statistical power design, which they leveraged to justify their statistics with a smaller sample size compared to the original one. | We used a univariate and multivariate logistic regression model to look for any association between TB and risk factors such as age, gender, BMI, diabetes, hypertension, history of TB tobacco smoking, alcohol consumption and cooking fuel used in the household.  We made use of the algorithm suggested by Van Wijngaarden-Dekker-Brent algorithm (Brent, 1973) to test the statistical power of the regression models which are more than 80% as required to ensure the statistical significance. |
